# Speckle Noise Removal Model Based on Diffusion Equation and Convolutional Neural Network

**DOI:** 10.1155/2022/5344263

**Published:** 2022-06-15

**Authors:** Siwei Nao, Yan Wang

**Affiliations:** ^1^Medical Technology School, Qiqihar Medical University, Qiqihar 161000, China; ^2^Basic Medical Science School, Qiqihar Medical University, Qiqihar 161000, China

## Abstract

The image denoising model based on convolutional neural network (CNN) can achieve a good denoising effect. However, its robustness is poor, and it is not suitable for direct noise removal tasks. Differently, the image denoising method based on the diffusion equation is more stable and has theoretical guarantees. In order to give full play to the advantages of CNN and diffusion equation in image denoising, this paper proposes a speckle noise denoising model via a combination of the two tools. Firstly, based on the mathematical model of speckle noise, a class of neural network speckle noise removal model which mixes residual learning and structure learning is proposed using image decomposition theory. Then, in order to solve the hyperparameter problem that the model depends on noise variance, a noise variance estimation algorithm based on a nonlinear diffusion equation is proposed. Finally, a speckle noise denoising model based on diffusion equation and CNN is obtained. Numerical simulation experiments verify the accuracy of the variance estimation algorithm and also the denoising effect and practical application value of the proposed method.

## 1. Introduction

In recent years, imaging technology has continued to develop and has been widely used in aerospace, geological remote sensing, and digital medicine. In the field of digital medicine, coherent imaging systems play a crucial role, especially the ultrasound imaging Taxt [1]. The principle of ultrasound imaging technology is to use ultrasound to scan tissues and organs in the human body and then collect and process reflected echo signals to obtain ultrasound images. Compared with other medical imaging technologies such as CT and MRI, ultrasound imaging has the advantages of being harmless to the human body and can be realized in real time. So, it can be widely used in clinical practice. However, due to the coherent characteristics of the ultrasonic imaging system, the scattered echoes of the ultrasonic technique may interfere and generate speckle noise, which reduces the signal-to-noise ratio (SNR) of the image and seriously affects the subsequent image postprocessing works. Therefore, it is very necessary to study the technology of speckle noise removal in ultrasound images.

In practical clinical applications, the logarithmic compression technology is used to compress the original echo signal to a suitable range, and the contrast of the signal is adjusted to enhance the darker details [2]. Loupas et al. [3] proposed the mathematical model of ultrasound image noise as follows:

(1) $$\begin{array}{c} f=u+\sqrt{u}N, \end{array}$$

where *u* is the real image, *f* is the noise image, and *N* is the Gaussian white noise with mean 0. This noise model is used in many ultrasound image denoising models. Loupas et al. [3] proposed the adaptive weighted median filtering, and Dutt and Greenleaf [2] proposed adaptive unsharp mask filtering based on local statistics per pixel. At the same time, the model based on wavelet analysis was also applied to the speckle denoise problem of compressed ultrasound images. Abrahim and Kadah [4] proposed a speckle noise removal model combining wavelet shrinkage and TV regularization to protect the boundaries and details of the image. Barcelos and Vieira [5] used the boundary control function to detect high-level noise near the noise point to improve the fidelity term and proposed an adaptive boundary control variational model. After that, Krissian et al. [6] proposed the following fidelity term of the speckle noise removal model for ultrasound images:

(2) $$\begin{array}{c} E\left(u\right)=\int_{\Omega }\frac{{\left(f-u\right)}^{2}}{u}\text{dx}. \end{array}$$

In 2011, Jin and Yang [7] proposed the following minimization function:

(3) $$\begin{array}{c} \underset{u\in BV\left(\Omega \right)}{\text{argmin}}\left\{\int_{\Omega }\left|Du\right|\text{dx}+\lambda \int_{\Omega }\frac{{\left(f-u\right)}^{2}}{u}\right\}\text{dx}. \end{array}$$

In this work, Jin and Yang also demonstrated the theoretical properties of the model in (3). Since then, there were also some corresponding improved models, such as Barcelos and Vieira [5] and Hacini and Djemal [8, 9]. In recent years, the ultrasonic image denoising model based on partial differential equations has been newly developed. Zhang et al. [12] proposed a speckle noise removal method based on nonlinear diffusion equation in 2009. In 2014, Bhateja et al. [10] proposed a weighted diffusion filtering method and its improved model. In 2018, Zhou et al. [11] proposed a variable exponential diffusion equation for speckle noise removal in ultrasound images, described as follows:

(4) $$\begin{array}{c} \frac{\partial u}{\partial t}=di\;v\left(\frac{\nabla u}{1+{\left(\left|\nabla u_{\rho }\right|/K\right)}^{\beta \left(u\right)}}\right),\left(x,t\right)\in \Omega \times \left(0,T\right),\\ \frac{\partial u}{\partial \overset{⟶}{n}}=0,\left(x,t\right)\in \partial \Omega \;\times \left(0,T\right),\\ u\left(x,0\right)=f\left(x\right),x\in \Omega . \end{array}$$

In equation (4), *K*, *ρ* > 0, *u*_*ρ*_=*G*_*ρ*_*∗u*, *β*(*u*)=2 − 2|*u*|^*α*^/*M*^*α*^+|*u*|^*α*^. The main idea is to change the diffusion type of different image feature regions through variable exponential function.

Since the 1990s, there have been many research results based on supervised learning in machine learning in the field of image processing. The main idea is to extract features from existing data to train the corresponding machine learning model [13]. The key part of the algorithm based on machine learning is to select which features in the image to represent the image, and traditional machine learning algorithms generally extract features manually. However, due to the limited features extracted manually, its image processing effect also has certain limitations. Deep learning algorithms, which have been highly praised in recent years, try to let computers automatically learn optimal features from the current datasets. Among them, the most representative of deep learning is the convolutional neural network (CNN), which automatically finds features in images through multiple convolutional layers. In 1980, Fukushima [14] did some CNN-related research, and in 1995, Lo et al. [15] applied CNN to image processing. In 1998, Lecun et al. [16] proposed LeNet for digit handwritten recognition after the initial success in the last century because the training process of CNN requires a lot of computation, but the computer performance was limited at that time. It was not until 2012 that AlexNet proposed by Krichevsky et al. [17] won the ImageNet Challenge that year with a great advantage, and deep CNN once again attracted great attention. After that, deep learning developed rapidly, and many new models appeared, such as VGG [18] and ResNet [19]. Let *x*_in_, *x*_out_ denote the input and output of a neuron in the model, respectively, *w* and *b* are the learned parameters, *h*(·) is nonlinear activation functions, and then *x*_out_ can be expressed as follows:

(5) $$\begin{array}{c} x_{\text{out}}=h\left(w^{T}x_{in}+b\right). \end{array}$$

A neural network consists of several neurons stacked into multiple layers, and information is propagated between layers. When the propagation direction is only forward propagation without feedback connection, the neural network is also called feedforward neural network. The first layer of a feedforward network is called the input layer, the last layer is called the output layer, and the middle layer is called the hidden layer. When the neural network has a very large number of hidden layers, it is also called a deep neural network, such as Stacked Autoencoders (SAEs), Restricted Boltzmann Machines (RBMs), and CNN.

At the same time, there are many algorithms that apply neural networks to the field of image restoration. One of the representative research results is the CNN model for image denoising. Jain and Seung [20] proposed a CNN image denoising framework, which achieved good results in both nonblind denoising and blind denoising. Vincent et al. [21] proposed stack sparse denoising. Harmeling [22] proposed a multilayer perceptron-based denoising network structure. In 2017, Zhang et al. [23, 24] used deep convolutional neural network (DnCNN) for residual image learning and then obtained denoised images. The two key points of the DnNN network are residual learning [19] and batch normalization. In the training process of the DnCNN model, the mean square error between the predicted residual image and the real residual image is used as the loss function of the model. The symmetric convolutional autoencoder proposed by Mao et al. [25] is composed of multiple convolutional layers and deconvolutional layers, which learns from noisy image to restore clean image from one end to other end. RED-Net extracts the features of the image through the convolution layer and avoids the influence of noise, and the subsequent deconvolution layer reconstructs the image according to the obtained features. Different from DnCNN, the loss function in RED-Net is chosen as the mean squared error between the predicted image and the real image.

Although deep learning has achieved good experimental results in image restoration, the model is less robust and lacks a sound theoretical explanation. At present, many scholars try to use mathematical theory to theoretically explain the deep learning framework. Many studies have shown that there is a certain connection between deep learning and partial differential equations [26]. For example ResNet can be interpreted as a specific differential equations [27].

This paper proposes a speckle noise removal model for ultrasound images based on diffusion equation and CNN. In Section 2, based on image decomposition theory, a hybrid CNN speckle noise removal model is proposed, which is divided into two parts: image noise estimation subnetwork and image structure estimation subnetwork. In Section 3, an estimation algorithm of speckle noise level in ultrasound images based on diffusion equation is proposed and used as a parameter of denoising model based on diffusion equation and CNN. Finally, in Section 4, the model proposed in this paper is numerically realized, and some experimental results are given. And the experimental results demonstrate the effectiveness of the model in this paper.

## 2. Image Speckle Denoising Model Based on Hybrid CNN

According to the mathematical model of speckle noise, the variance of speckle noise in ultrasound images depends on the gray value of the image. In the area with a large gray value of the image, the noise variance is large, and in the area with a small gray value of the image, the noise variance is small. From a local point of view, when the fluctuation of the gray value of the original image is relatively smooth, the noise level is relatively similar. According to image decomposition theory, images can generally be decomposed into two parts: cartoon part and texture part [28]. The cartoon part contains the main structure and the gradually changing part of the image, showing a large smooth area. So its gray value is relatively close, and then it can be seen from the noise model that the variance of the noise fluctuates less. The texture part contains the small-scale detail information and noise of the image. According to the noise model, this part of the noise fluctuates greatly.

**Figure 1:**
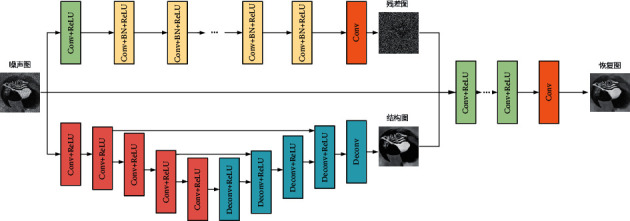
Schematic diagram of the hybrid CNN speckle noise removal model.

Denoising algorithms based on convolutional neural networks have made good progress in additive denoising. Zhang [23] proposed the DnCNN model, the main idea of which is to learn the noise of the image and subtract the noise image from the learned noise to obtain a noise-free image. Shen et al. [25] proposed the self-encoding structure RED-Net in the literature [25], which gradually compresses the noisy image information through convolutional layers and filters the noise information while retaining the structural information for the subsequent image reconstruction process. Based on the image decomposition theory, this paper combines the above two types of network structures, uses DnCNN to estimate the texture part of the noisy image, and uses RED-Net to estimate the cartoon part of the noisy image. A hybrid CNN is proposed to build a speckle noise removal model. The input of the model is a noise image, and the output is a restored noise-free image. As shown in Figure 1, the model is divided into two subnetworks: an image noise estimation network and an image structure estimation network. The image noise estimation network is a fully convolutional network. The image structure design network is a skip-connected convolutional autoencoder. Finally, the two-part network obtains the output predicted image through multiple convolutional layers. The advantage of this network is that, based on the image decomposition theory, CNN is used to simultaneously remove noise and extract image structural features and finally integrate the noise information and structural information to obtain the predicted image.

In Figure 1, Conv is the convolution layer, Deconv is the deconvolution layer, BN is the batch regularization, and ReLU is the linear rectification activation function. Next, let *f* be the noise image, $\hat{\mu }$ be the image noise estimate, and $\hat{s}$ be the image structure estimate. For the image noise estimation subnetwork, let Ψ_*R*_(Θ_*R*_, ·) be the learned mapping, where Θ_*R*_ is the parameter to be learned, then the input of the network is the noise image *f*, and the output is the estimation of image noise $\hat{\mu }$, which satisfies

(6) $$\begin{array}{c} \hat{\mu }=\Psi_{R}\left(\Theta_{R},f\right). \end{array}$$

As shown in Table 1, the noise estimation subnetwork is a fully convolutional network with 17 layers of convolution, the size of the convolution kernel is 3 × 3, the stride is 1, and appropriate zero padding is done to ensure that each layer outputs an image has the same length and width as the input image. The first 16 convolutional layers of the network all need the ReLU activation function. The 2–16 convolutional layers have batch regularization, the number of feature maps output by the middle 1–16 layers is 64, and finally, a new image is obtained that is the same size as the original image.

In the process of designing the CNN structure, the loss function is a crucial part, and its role can be analogous to the energy functional in the traditional method. Different loss functions should be designed for different image processing tasks, such as the Softmax function in the commonly used image classification network and *L*^1^ and *L*^2^ regression in the target detection network. For the image denoising problem, the commonly used loss function is the *L*^2^ norm or *L*^1^ norm between the restored image and the noisy image. In order to make the parameters in the two subnetworks fully trained, the loss function of the model also includes the direct constraints of the two subnetworks. The loss function used in the hybrid CNN model proposed in this paper is

(7) $$\begin{array}{c} L\left(\Theta \right)=\frac{1}{N}\sum_{i=1}^{N}{\left\|\Psi_{R}\left(\Theta_{R},f_{i}\right)-\left(f_{i}-u_{i}\right)\right\|}^{2}+{\left\|\Psi_{S}\left(\Theta_{S},f_{i}\right)-u_{i}\right\|}^{2}+{\left\|\Psi \left(\Theta ,f_{i}\right)-u_{i}\right\|}^{2}, \end{array}$$

where (*f*_*i*_, *u*_*i*_)_*i*=1_^*N*^ is the *N* noisy images and no-noise images in the training set, Ψ(Θ, ·) is the mapping learned by the entire network, and Θ represents all parameters in the network.

**Figure 2:**
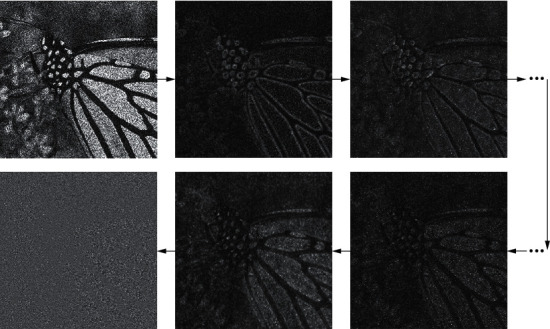
Processing of image noise estimation subnetwork.

**Figure 3:**
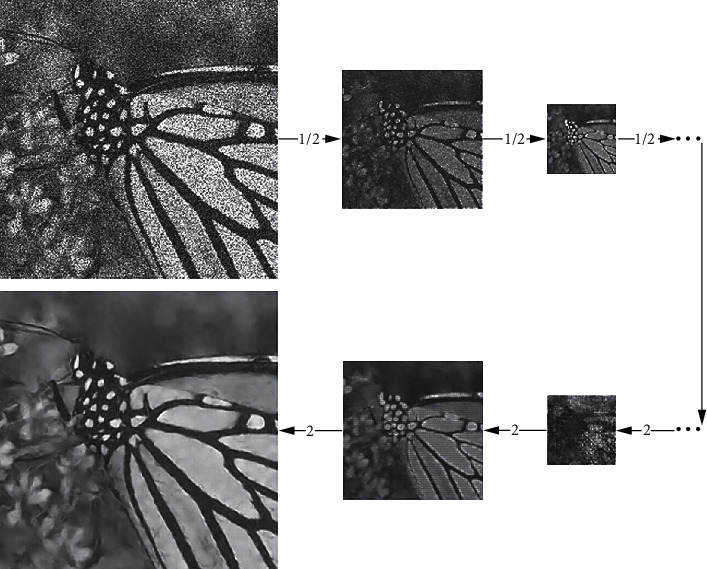
The processing of the image structure estimation subnetwork, 1/2 and 2 are the multiples of image scaling.

Figure 2 shows the processing of a noisy image by the image noise estimation subnetwork in the hybrid CNN denoising model. The main purpose of the noise estimation subnetwork is to estimate the noise of the image. With the progressive number of network layers, the structural information in the image becomes less and less, and the noise information becomes more and more.  Figure 3 shows the processing process of the image structure estimation subnetwork. The image information is gradually compressed by convolution downsampling. The structural information of the image is extracted, the noise information is removed, and the detailed information of the image is gradually restored in the process of upsampling.

## 3. Image Speckle Denoising Model Based on Diffusion Equation and CNN

**Figure 4:**
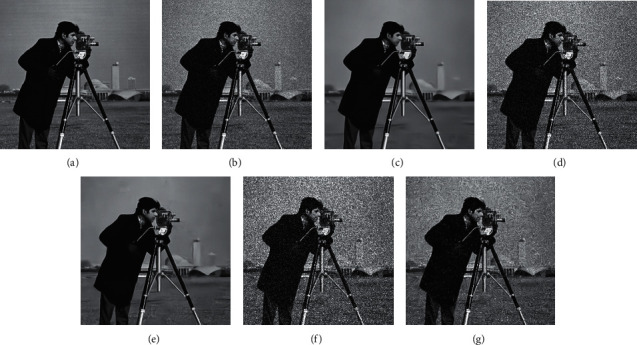
Restoration results of DnCNN on images with different noise variances. (a) The original image, (b) noise: *σ* = 15, (c) restored image, (d) noise: *σ* = 25, (e) restored image, (f) noise: *σ* = 35, and (g) restored image.

CNN can achieve very good experimental results in image restoration, but it has a fatal disadvantage: the dependence on the noise variance parameter seriously limits its application in practical engineering problems. DnCNN and RED-Net need to be consistent in the image noise in the training process dataset, so these network structures can remove noise with known variance well, but they will not get a good denoising effect for images with unknown noise variance.  Figure 4 shows a set of experimental results, trained on the training dataset with noise level *σ* = 25, on DnCNN and tested on noise image with variances of 15, 25, and 35, respectively. From Figure 4, it can be found that, for the noise image with a noise variance of 15, the restoration result of DnCNN is too smooth, and the detailed information is lost. For the noise image with a noise variance of 35, the noise removal is insufficient, and there is still a lot of noise in the restoration result. For an image with a noise variance of 25, the denoising effect is very good; that is, the noise is removed, and the details are retained. In the actual application process, the variance of image noise is usually unknown, so directly using DnCNN to denoise may not necessarily get a good denoised image, which seriously limits the value of the neural network model in practical engineering applications. The same problem exists with the hybrid CNN model proposed in the previous section.

Aiming at the problem that the above CNN model relies on the noise variance, an intuitive outcome is to use noise images with different noise variances as the training dataset, that is, mixed noise images. However, this has caused a series of problems; for example, the training process is more difficult, and the recovery effect of the final model is generally worse.  Table 2 shows the PSNR results of the DnCNN model trained with different training sets. It can be found that, under different noise levels, the PSNR of the mixed case is lower than that of the single case.

Another method used in this paper to solve this problem is to predict the noise variance of noisy images in advance. The specific method is to use the diffusion equation-based ultrasonic image denoising model [11] to estimate the noise level. The model is not sensitive to noise variance due to the existence of Gauss convolution. Among the three key parameters (*β*, *K*, *α*) in the model, generally, *β* = 1, *K* = 1, and *α* varies between 1 and 2 with the noise level. But in fact, when the parameters *α* are selected within [1, 2], the impact on the optimal results of the model is far less than the impact of noise variance on neural network models. The specific nonlinear diffusion equation ultrasonic image speckle noise variance estimation method is given in the following.

**Step 1 .**

Diffusion equation preprocessing.

Here, *f* is the noise image, *u* is the noise-free image, and *u*_*pre*_ is the preprocessed image obtained by nonlinear diffusion equation. According to the work by Zhou et al. [29], *u*_*pre*_ can be handled accordingly.

**Step 2 .**

Noise variance estimation region selection.

Using the region detection operator in Guo et al. [12],

(8) $$\begin{array}{c} \beta \left(x\right)=2-\frac{2}{1+{\left|\nabla G_{\sigma }*u_{\text{pre}}\right|}^{2}}. \end{array}$$

The image structure *u*_*pre*_ in the preprocessing result Ω is detected. The image area is divided into two categories $\tilde{\Omega }=\left\{x\in \Omega ,0\leq \beta \left(x\right)<2\right\}$ and $\hat{\Omega }=\left\{x\in \Omega ,0\leq \beta \left(x\right)<2\right\}$.

**Step 3 .**

Noise variance estimation.

The sample variance of the noisy image *f* is calculated as

(9) $$\begin{array}{c} s_{i}^{2}=\frac{1}{n_{i}-1}\sum_{x\in \Omega_{i}}{\left(f\left(x\right)-m_{i}\right)}^{2}, \end{array}$$

where *n*_*i*_ is the number of pixels in the subregion Ω_*i*_ and *m*_*i*_ is the mean of the noise image *f* in this region:

(10) $$\begin{array}{c} m_{i}=\frac{1}{n_{i}}\sum_{x\in \Omega_{i}}f\left(x\right). \end{array}$$

To sum up, the specific process of the speckle noise denoising model for ultrasound images based on the diffusion equation and CNN proposed in this paper is as follows: first, the noise image is preprocessed by the nonlinear diffusion equation so as to estimate the noise variance. Denoising the image is treated as a parameter of the hybrid CNN model and taking the noisy image as input. It is worth mentioning that the whole process does not require any input parameters, thus improving the value of the model in practical applications.

## 4. Experimental Results and Analysis

**Figure 5:**
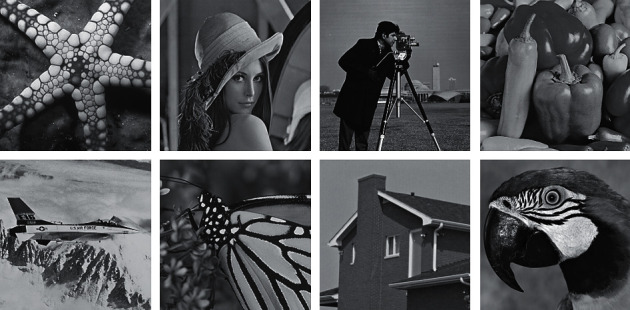
Test image.

This part presents the experimental results of the model in this paper and compresses it with other models. The models compared with the model in this paper are the VA model [7], SRAD model [31], DnCNN, and RED-Net [25]. It is worth noting that the parameters of all models are adjusted to the optimal parameters according to the original paper, and the deep learning models are trained using the same training dataset. The optimization method used in the training process of the hybrid CNN model is ADAM [32]. The learning rate is 0.001, the number of batch training images is 128, and the number of training times is 50.  Figure 5 is the test image of the experiments in this section.

**Figure 6:**
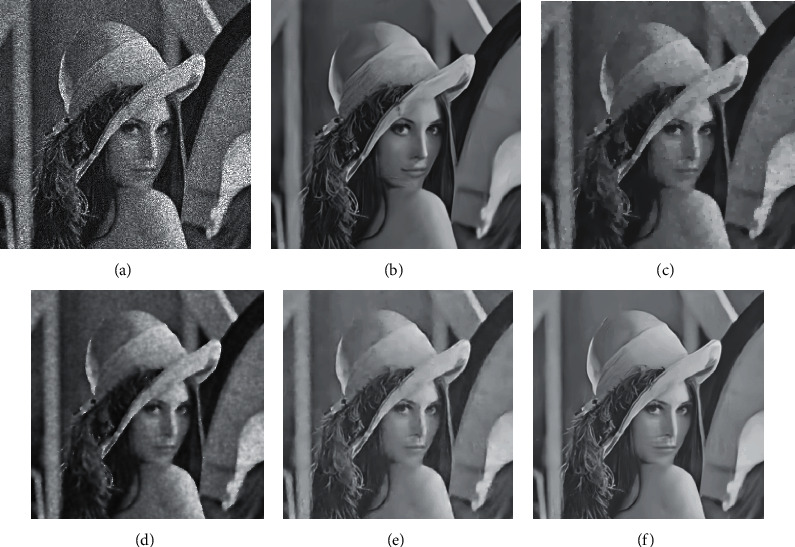
Results of test image II. (a) *σ*=2. (b) The results of this model. (c) VA results. (d) SRAD results. (e) RED-Net results. (f) DnCNN results.

**Figure 7:**
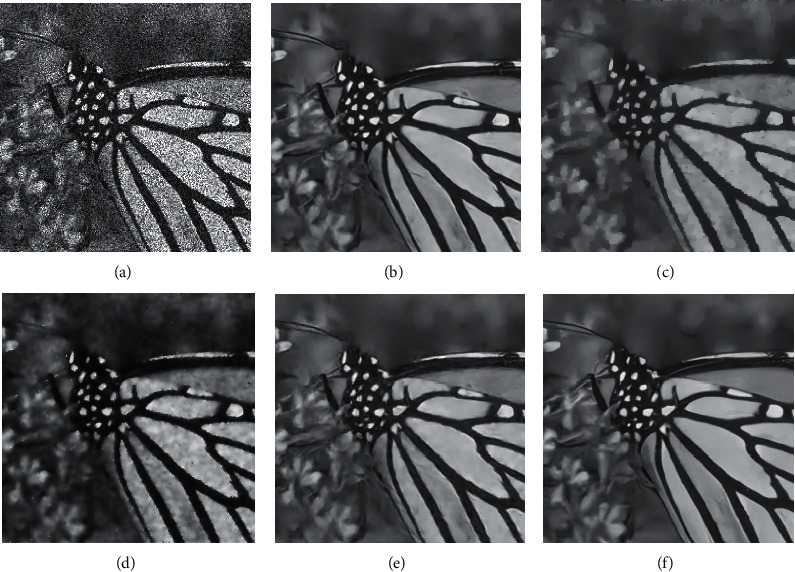
Results of test image VI. (a) *σ*=4. (b) The results of this model. (c) VA results. (d) SRAD results. (e) RED-Net results. (f) DnCNN results.

Table 3 gives the PSNR values of each model under different noise levels for 8 test images. In three sets of noise level experiments, the algorithm based on neural network is far superior to the traditional method in terms of PSNR index, which is 3 dB higher on average. It is worth mentioning that the PSNR value of the model in this paper is higher than other methods in most cases. Figures 6 and 7 show two sets of test result images. From the perspective of visual effects, although the traditional method can remove noise, the damage to the details of the image is very serious. The algorithm based on deep learning can not only achieve higher PSNR but also have better visual effects. The visual effects of the model in this paper are outstanding. Although the model in this paper and the DnCNN model are very similar in terms of visual effects, there are certain differences in careful observation. For example, for the structure of the hat spike and mouth in Figures 6(b) and 6(f), it can be found that the image restored by the model in this paper is more detailed and closer to the original image. At the same time, a similar phenomenon can be found by comparing the petals in Figures 7(b) and 7(f). The results illustrate the advantages of our model in speckle noise removal.

**Figure 8:**
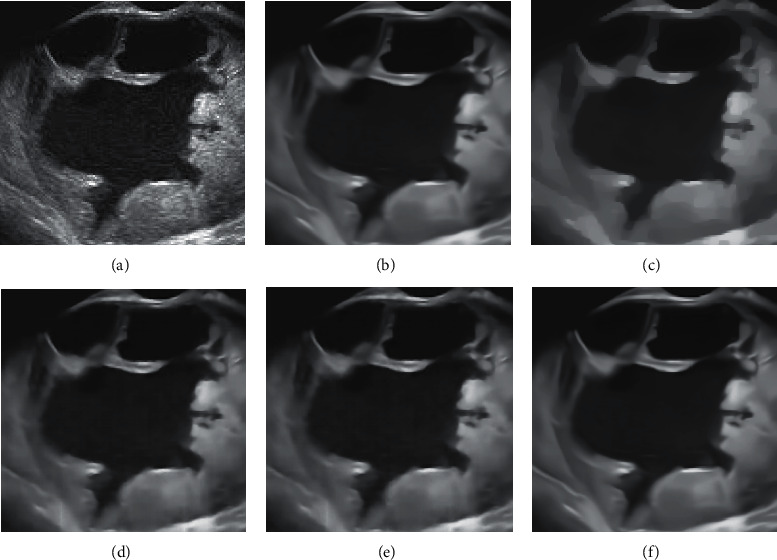
Results of real ovarian cancer ultrasound images. (a) The original image. (b) The results of this model. (c) VA results. (d) SRAD results. (e) RED-Net results. (f) DnCNN results.

Table 4 presents the experimental results of speckle noise variance estimation. Eight test pictures are tested, the noises *σ*=4,9,16 are added to the test pictures, respectively, and then the noise level estimation method proposed in this paper is used to estimate the noise. It can be seen from Table 4 that, for different noise levels and different test images, the model proposed in this paper can predict the noise variance well, and the average error is about 0.06. This group of experiments verifies the effectiveness of the noise level estimation method in this paper. Finally, the proposed model is applied to noise removal in real ultrasound images.  Figure 8 presents a set of experimental results, from which it can be seen that, compared with the traditional method and other neural network methods, the model in this paper can better restore the detailed information in the image and smooth the noise, which is better than other models in terms of visual effect. The model in this paper uses the diffusion equation method to estimate the noise level, thus avoiding the influence of the noise level parameter on the model, and in practice, the noise level has a great influence on the experimental results.

## 5. Conclusion

In this paper, an ultrasonic image denoising model based on diffusion equation and CNN is proposed, which fully utilizes the advantages of diffusion equation and CNN in image denoising. Based on image decomposition theory, the characteristics of texture and cartoon parts in ultrasound images are analyzed, and a hybrid CNN image denoising model is proposed, which is divided into noise estimation subnetwork and structure estimation subnetwork. Afterward, aiming at the problem that the convolutional neural network model depends heavily on the noise level, a method for estimating the noise variance of ultrasound images is proposed in this paper by taking advantage of the insensitivity of the diffusion equation image denoising model to the noise level. The final experimental results show that the model in this paper can remove the noise in the image and restore the image structure information, and the model in this paper does not need any parameters and has great practical engineering application value.

## Figures and Tables

**Table 1:** Specific parameters of the network structure of the hybrid CNN speckle noise removal model.

Subnet type	Layers	Type	Number of input and output feature maps	Convolution kernel size and stride
Noise	1	Conv + ReLU	1⟶64	3 × 3,1
	2–16	Conv + BN + ReLU	64⟶64	3 × 3,1
	17	Conv	64⟶1	3 × 3,1
Structure	1	Conv + ReLU	1⟶64	3 × 3,2
	2–5	Conv + ReLU	64⟶64	3 × 3,2
	6–9	Deconv + ReLU	64(+64)⟶64	3 × 3,2
	10	Deconv	64⟶1	3 × 3,2
Output	1	Conv + ReLU	1 + 1⟶64	3 × 3,1
	2–6	Conv + ReLU	64⟶64	3 × 3,1
	7	Conv	64⟶1	3 × 3,1

**Table 2:** PSNR results of DnCNN on different training sets.

Test image	I	II	III	IV	V	VI	VII	VIII	Average
Noise level *σ* = 15									
DnCNN (single) DnCNN (mix)	32.2732.01	34.64	32.62	33.37	31.72	33.29	35.02	33.39	33.29
		34.22	32.11	33.12	31.54	32.93	34.83	33.01	32.97
Noise level *σ* = 25									
DnCNN (single) DnCNN (mix)	29.51	32.48	30.28	30.92	29.43	30.48	33.36	30.89	30.91
	29.30	32.12	29.34	30.12	29.08	30.21	33.01	30.34	30.44

**Table 3:** PSNR results of different models on test images.

Test image	I	II	III	IV	V	VI	VII	VIII	Average
Noise level *σ* = 2									
Proposed	**29.74**	**31.89**	**31.00**	**31.67**	**29.31**	**31.30**	**33.64**	**30.59**	**31.14**
SARD	26.15	28.43	27.93	28.10	26.43	28.53	30.01	27.71	28.23
VA	26.32	28.96	28.01	28.31	26.77	28.69	30.28	27.82	28.51
DnCNN	29.65	31.88	30.95	31.66	29.23	31.24	33.63	30.57	31.10
RED-net	28.51	30.39	29.14	29.97	28.07	29.73	32.07	29.14	29.63
Noise level *σ* = 3									
Proposed	**27.42**	**30.03**	**29.10**	**29.66**	**27.30**	**29.37**	**32.05**	**28.79**	**29.21**
SARD	26.78	27.54	26.07	25.78	23.12	25.65	27.51	24.17	25.61
VA	24.11	25.74	25.48	25.98	23.77	25.87	27.98	24.76	25.91
DnCNN	27.36	29.85	29.09	29.64	27.14	29.30	31.94	28.77	29.14
RED-net	26.75	28.87	27.27	28.28	26.43	28.20	30.39	27.78	28.06
Noise level *σ* = 4									
Proposed	**25.82**	**28.43**	27.72	**28.39**	**25.97**	**27.88**	**30.58**	**27.52**	**27.78**
SARD	23.13	25.41	24.83	25.51	23.17	24.82	27.32	24.76	25.01
VA	23.51	25.62	25.14	25.86	23.67	25.21	27.88	25.03	25.62
DnCNN	25.65	28.33	**27.78**	28.12	25.81	27.62	30.40	27.48	27.64
RED-net	30.31	27.94	27.08	27.61	25.48	27.05	29.53	27.00	27.14

**Table 4:** Noise variance estimation results on different test images.

Test image	I	II	III	IV	V	VI	VII	VIII	Average error
*σ* = 4	4.125	4.027	4.039	4.031	3.945	4.042	3.994	4.028	0.044
*σ* = 9	9.120	9.029	9.052	9.063	9.158	9.061	9.016	8.966	0.066
*σ* = 16	15.901	16.057	16.101	16.112	15.867	16.073	16.026	16.059	0.082

## Data Availability

The dataset can be accessed upon request.
